# Epstein-Barr Virus and multiple sclerosis in a Spanish cohort: A two-years longitudinal study

**DOI:** 10.3389/fimmu.2022.991662

**Published:** 2022-09-14

**Authors:** María Inmaculada Domínguez-Mozo, Lorena López-Lozano, Silvia Pérez-Pérez, Ángel García-Martínez, María José Torrejón, Rafael Arroyo, Roberto Álvarez-Lafuente

**Affiliations:** ^1^ Grupo Investigación de Factores Ambientales en Enfermedades Degenerativas, Instituto de Investigación Sanitaria del Hospital Clínico San Carlos (IdISSC), Madrid, Spain; ^2^ Servicio de Análisis Clínicos, Instituto de Medicina del Laboratorio, Hospital Clínico San Carlos, Madrid, Spain; ^3^ Servicio Neurología, Hospital Universitario Quirónsalud Madrid, Madrid, Spain

**Keywords:** multiple sclerosis, Epstein-Barr virus, EBNA-1, VCA, HLA, GWAS, EOMES, vitamin D

## Abstract

**Objectives:**

1. To analyze the prevalence and levels of anti-EBNA-1 and anti-VCA IgG antibodies of Epstein-Barr virus (EBV) in a Spanish cohort of multiple sclerosis (MS) patients and their interactions with other environmental and genetic risk factors. 2. To analyze the association of the evolution of these antibodies with the clinical response to different disease modifying therapies (DMTs) after two-years of follow-up. 3. To assess their possible correlation with the class II HLA alleles as well as with several SNPs identified in GWAS related to disease susceptibility.

**Materials and methods:**

We recruited 325 MS patients without DMT (serum samples were collected 1-3 months before starting a therapy) and 295 healthy controls (HC). For each patient we also collected serum samples 6, 12, 18 and 24 months after starting the DMT. EBNA-1 and VCA IgG titers were analyzed by ELISA; 25(OH)D levels were analyzed by immunoassay; HLA DRB1*15:01 allelic variant was analyzed by Taqman technology.

**Results:**

1. 97.8% (318/325) vs. 87.1% (257/295) positives for EBNA-1 in MS patients and HC, respectively (p<0.0001; O.R. = 6.7); 99.7% (324/325) vs. 94.6% (279/295) for VCA in MS patients and HC, respectively (p=0.0001; O.R. = 18.6). All MS patients were positive for EBNA-1 and/or VCA IgG antibodies vs. 280/295 (94.9%) HC (p<0.0001). IgG titers were also significantly higher in MS patients than in HC. 2. We did not find any statistical correlation in the variation of the EBNA-1 and VCA IgG titers between baseline and 24 month visits with the number of relapses, progression, clinical response, NEDA-3 condition or therapeutic failure. 3. When we compared different epidemiological and clinical variables between those with genetic factors associated with lower EBNA-1 IgG titers and all other MS patients, we found MS started 3.5 years later among the first.

**Conclusions:**

These results confirm that MS occurs rarely in absence of EBV. An intriguing association between genetic burden and lower EBNA-1 IgG titers was associated with an earlier age of disease onset. Similar studies with B-cell–targeted therapies should be performed.

## Introduction

Multiple sclerosis (MS) is a neurological chronic inflammatory disease of the central nervous system (SNC) characterized by demyelination and axonal destruction in different degrees (1). Although the etiology of MS is still unknown, increasing evidences show that environmental factors could have a role in the disease. Among the related environmental factors, the infection by certain viruses have been associated with the development of MS (2), especially the Epstein-Barr virus (EBV) (3–5). Furthermore, it has been recently published that EBV could be the leading cause of MS (6); in this study, authors found that risk of MS increased 32-fold after infection with EBV but was not increased after infection with other viruses.

Different disease modifying therapies (DMTs), such as interferon-beta (IFN-beta), glatiramer acetate (GA) or natalizumab (NTZ) are able to reduce the relapse rate and the rate of disability progression (7–9). Since DMTs are able to change the evolution of the disease, a relation between the clinical response to these DMTs and the viruses involved in MS could be demonstrated. Moreover, different genetic factors as human leukocyte antigen (HLA) allelic variants and other genetic variants identified by genome wide association studies (GWAS) are related to MS susceptibility (10). However, a significant proportion of MS heritability remains unexplained. Different explanations for the missing heritability in MS have been proposed, including gene-environment interactions.

Thus, the objectives of this study were: 1. To analyze the prevalence and levels of anti-EBNA-1 and anti-VCA IgG antibodies in a Spanish cohort of MS patients and their possible interactions with other environmental factors such as smoking habit and vitamin D and with the MS genetic risk factor HLA-DRB1*15:01. 2. To analyze the possible association of the evolution of the anti-EBNA-1 and anti-VCA IgG antibody titers with the clinical response to different disease modified therapies (DMTs) after two-years of follow-up. 3. To assess the possible correlation of the anti-EBNA-1 and anti-VCA IgG antibody titers with the class II HLA alleles as well as with several SNPs identified in GWAS related to disease susceptibility.

## Materials and methods

### Design

This is a retrospective study. Inclusion criteria: MS patients over 18 years old diagnosed by Poser (11) or 2010 McDonald (12) criteria with: (1) interferon-beta (IFN-beta), glatiramer acetate (GA) or natalizumab treatment for at least two years; (2) serum samples collected within a month before treatment onset and 24 months after treatment initiation; (3) expanded Disability Status Scale (EDSS) score at DMT onset and two years later, (4) number of relapses since the beginning of the disease and during the first two years of treatment; (5) an analysis of antibodies against IFN-beta in MS patients treated with this DMT. Exclusion criteria: pregnant woman and MS patients with secondary progressive or primary progressive diagnosis. A cohort of healthy controls was also included in the study to be compared with those MS patients before starting DMT; the inclusion criteria were: blood donors with more than 18 years old. Exclusion criteria for healthy controls: pregnant woman, first and second degree relatives with MS or with any other autoimmune disease.

### Patients

Patients belonged to the following hospitals: Hospital Clínico San Carlos (Madrid) and Hospital Universitario Quirónsalud Madrid. Neurologists of the Multiple Sclerosis Units of those hospitals collected all the clinical data. Blood donors belonged to Hospital Clínico San Carlos.

### Ethics statement

This study was approved by local Ethic Committees of the following centers: Comité Ético de Investigación Clínica del Hospital Clínico San Carlos and Comité de Ética de la Investigación del Hospital Fundación Jiménez Díaz (for patients recruited at Hospital Universitario Quirónsalud Madrid). A written informed consent was received and signed for each one of the MS patients and controls recruited. All experiments were performed in accordance with relevant guidelines and regulations.

### Response criteria

Progression was defined depending on pre-treatment Expanded Disability Status Scale (EDSS) score: 1) increase ≥1.5 points at 24-months visit if pre-treatment EDSS=0; 2) increase ≥1 point at 24-months visit if pre-treatment EDSS was ≥1 and ≤5; 3) increase ≥0.5 points at 24-months visit if pre-treatment EDSS was ≥5.5. We considered relapses as a worsening of neurological damage or a new symptom or other abnormality referable to MS; they should last at least 24 hours with a subsequently period of stability of at least one month. One month prior DMT onset was performed a magnetic resonance imaging (MRI) of the brain in 1.5T scanners following a previous published protocol (13); later MRIs were performed one and two years after starting these therapies. We collected the following sequences for this study: T1-weighted imaging with Gd enhancement, axial fluid-attenuated inversion recovery (FLAIR) T2, axial T2-weighted imaging and axial proton density T2-weighted imaging. To cover the entire brain we acquired slice thickness of 5 mm to analyze contiguous axial sections. With the previous definitions, the following response criteria were established after two years of follow-up: clinical response (defined as an absence of relapses and disability progression), therapeutic failure (≥2 relapses and/or disability progression), and NEDA-3 (no evidence of disease activity: no relapses, no disability progression, with no new T2 lesions or Gd+ lesions).

### Researched variables

We analyzed the following variables in the MS group:

- Demographic, clinical and radiological: gender, age at disease onset, age at DMT onset, EDSS at DMT onset, progression of the disease after two-years of treatment, disease duration, number of relapses two-years after DMT onset and annualized relapse rate since the beginning of the disease, number of T2 and Gd+ lesions in the MRI performed at recruitment and 1 and 2 years after DMT onset.
- Genetic: Class II HLA alleles (including HLA DRB1*15:01 allelic variant), and SNPs related to MS susceptibility selected from GWAS (**Supplementary Table 1**).
- Environmental: IgG antibody response to EBV (EBNA-1 and VCA IgG titers) in baseline serum samples, as well as their changes between the baseline and 24 month sample. Smoking habit (current smoker or not smoker) and 25(OH)D levels at baseline sample prior DMT onset.
The following variables were analyzed in the healthy control group:
- Demographic: gender and age at sample collection.
- Genetic: HLA DRB1*15:01 allelic variant.
- Environmental: IgG antibody response to EBV (EBNA-1 and VCA IgG titers), smoking habit (current smoker or not smoker) and 25(OH)D levels at sample collection.

### Serum samples

For each patient and control we collected one dry tube with blood. After collection, blood was clotted by leaving it at room temperature for 30 minutes; later, clot was removed by centrifuging 10 minutes at 1,500xg in a refrigerated centrifuge. Lastly, serum samples were aliquoted, and aliquots were frozen at -80°C.

### EBNA-1 and VCA IgG ELISA

Serum samples of MS patients and controls were tested with two tests of Trinity Biotech Captia™ (Bray, Co. Wicklow, Ireland), for the detection and quantification of anti-EBNA-1 and anti-VCA (BFRF3 antigen) IgG titers following manufacturer instructions, in an automated ELISA processing system (DS2, Dynex Technologies, USA). Results were expressed in arbitrary units (AU); they were calculated by multiplying the index value by 10 (the index value for each one of the samples = sample absorbance/cut-off value). Standard samples were run on each plate (the inter-assay coefficient of variation was under 5%). Samples from MS patients and healthy controls were analyzed in duplicate for each test. Those that were above 11 AU were considered positive and those that were below 9 AU were considered negative; doubtful samples, those that were between 9 and 11 AU were tested again.

### Detection of neutralizing antibodies (NAbs) against IFN-beta

In those RRMS patients that were under IFN-beta treatment we measured NAbs through the cytopathic effect (CPE) assay (14). Titers were calculated according to Kawade’s formula (15), and they were expressed in tenfold reduction unit (TRU). Positive samples were those with titers > 20 TRU/ml.

### 25(OH)D determination

25(OH)D levels were analyzed by immunoassay (Abbot, Wiesbaden, Germany), following the manufacturer’s instructions. To normalize the data due to the seasonal variation, we calculate the median value of each semester (MVS) for MS patients and healthy controls: 16.9 ng/ml for the first semester of the year and 24.0 ng/ml for the second semester of the year, in MS patients; 19.8 ng/ml and 24.7 ng/ml, respectively, in healthy controls. Finally, we performed two groups for MS patients and healthy controls: those with 25(OH)D levels>MVS and those with 25(OH)D levels<MVS.

### HLA genotyping

HLA Genotyping. Class II HLA-DR and -DQ was genotyped using SSOP technology (Sequence specific-oligonucleotid probe). HLA DRB1*15:01 allelic variant was analyzed by Taqman technology in a 7900HT Fast Real-Time PCR system, following manufacturer recommendations (Applied Biosystems, Foster City, CA, USA). Genotyping of different SNPs from GWAS was performed by iPLEX^®^ Gold MassARRAY Sequenom technology at the National Center of Genotyping (CEGEN. Valencia. Spain). These SNPs were included in this study because they were available as they had been analyzed previously in different genetic studies performed in our MS patients. In **Supplementary Table 1** are shown the SNPs included in this study.

### Statistical analysis

The chi-square or two-tailed Fisher’s exact test was used to test differences in categorical variables. Kruskall-Wallis analysis or the Wilcoxon rank-sum test was used to test differences in continuous variables. For the genetic study, allele and genotype frequencies were compared by the Chi-square test. We considered statistically significant differences when p<0.05; p-values were corrected for multiple comparisons with the Bonferroni method. Odds ratios (O.R.) and exact 95 percent confidence intervals (C.I.) were obtained with the following software: Epi Info v. 6.02 (CDC, Atlanta, USA) and SPSS Ver. 15.0 (SPSS Inc.). The graphs were created with GraphPad Prism^®^ 8.0.

## Results

### Patients eligible for the study and demographic characteristics of the population study

We recruited 325 relapsing-remitting MS (RRMS) patients that fulfilled all the inclusion criteria and 295 healthy controls (**Table 1**). Furthermore, from 246 MS patients and 295 healthy controls we had data from smoking habit; from 233 MS patients and 194 healthy controls we measured vitamin D levels at basal sample and at 24 month sample; from 315 MS patients and 270 healthy controls we assessed the HLA-DRB1*15:01 allelic variant; class II HLA-DR and -DQ were genotyped from 288 MS patients; 304 MS patients were genotyped for the SNPs shown at **Supplementary Table 1**; finally, from 215 MS patients we had MRI data.

**Table 1:** Demographical characteristics of the patients and healthy controls included in the study at the recruitment.

	MS patients	Healthy controls
Males	108	180
Females	217	115
**Previous treatments prior recruitment:**		
No previous treatments (naïve patients)	184	–
1 treatment	92	–
2 treatments	38	–
2 or more treatments	11	–
**Age at recruitment (years, med (P25-P75))**	36.0 (30.0-42.0)	39.0 (29.5-47.0)
**Age at disease onset (years, med (P25-P75))**	28.0 (24.0-34.0)	–
**Disease duration at recruitment (months, med (P25-P75))**	65.0 (18.0-118.0)	–
**EDSS at recruitment (med (P25-P75))**	2.0 (1.0-3.0)	–
**Annualized relapse rate since MS diagnosis (med (P25-P75))**	1.0 (0.6-1.7)	–
**Number of relapses 2 years before recruitment (med (P25-P75))**	2.0 (1.0-3.0)	–
**DMTs initiated at recruitment:**		
Glatiramer acetate	83 (53 naïve)	–
Interferon beta	131 (123 naïve)	–
Natalizumab	111 (8 naïve)	–
HLA DRB1*15:01 carriers (per (n/N))	38.1 (120/315)	16.3 (44/270)
**Smoking habit: smokers at recruitment (per (n/N))**	45.5 (112/246)	24.7 (73/295)

(med: median; P25, 25th percentile; P75, 75th percentile; EDSS, Expanded Disability Status Scale; per, percentage).

### Higher EBNA-1 and VCA IgG prevalence and titers in untreated MS patients than in healthy controls

We found statistical significant differences in the prevalence of EBNA-1 and VCA IgG antibodies between untreated MS patients (samples collected prior DMT onset) and healthy controls: 97.8% (318/325) vs. 87.1% (257/295) for EBNA-1 in MS patients and healthy controls, respectively (p=0.0000003; O.R. = 6.7); 99.7% (324/325) vs. 94.6% (279/295) for VCA in MS patients and healthy controls, respectively (p=0.0001; O.R. = 18.6). Furthermore, all MS patients were positive for EBNA-1 and/or VCA IgG antibodies vs. 280/295 (94.9%) healthy controls (p=0.00004). Regarding IgG titers, we also found statistical significant differences. The median value in MS patients was 25.4 AU for EBNA-1 and 59.9 AU for VCA vs. 24.2 AU for EBNA-1 and 55.5 AU for VCA in healthy controls (p<0.000001, for both IgG antibodies). Since major predisposing factors for MS, like HLA DRB1*15:01, vitamin D deficiency and smoking, have also been related with the IgG levels against EBV, we stratified according to them; comparisons between MS patients and healthy controls are shown in **Table 2**.

**Table 2:** Comparisons of EBNA-1 and VCA IgG titers and prevalence between untreated MS patients and healthy controls after stratification according to HLA DRB1*15:01 allele, smoking habit and levels of vitamin D.

	A. EBNA-1 IgG											
	HLA DRB1*15:01			NO HLA DRB1*15:01								
	**n/N (%)**		Titers*	**n/N (%)**			Titers*	**p (Chi)**		**OR (95% CI)**		**p (t-St.)**
MS**	119/120 (99.2)		25.5	189/195 (96.9)			25.2	n.s.		3.8 (0.4-84.3)		n.s.
**HC**	40/44 (90.9)		25.5	198/226 (87.6)			23.2	n.s.		1.4 (0.4-5.1)		n.s.
p***	0.006		0.004	0.0004			<0.00001					
**OR (95% CI)**	11.9 (1.2-288.1)			4.5 (1.7-12.3)								
	**CURRENT SMOKER**			**NO SMOKER**								
	**n/N (%)**		Titers*	**n/N (%)**			Titers*	**p (Chi)**		**OR (95% CI)**		**p (t-St.)**
MS**	110/112 (98.2)		25.3	134/134 (100)			25.5	n.s.		–		n.s.
**HC**	68/73 (93.2)		22.3	184/216 (85.2)			24.2	n.s.		2.4 (0.8-7.2)		n.s.
p***	n.s.		<0.00001	<0.00001			<0.00001					
**OR (95% CI)**	4.0 (0.7-31.1)			–								
	25(OH)D<MVS****			25(OH)D> MVS****								
	**n/N (%)**		Titers*	**n/N (%)**			Titers*	**p (Chi)**		**OR (95% CI)**		**p (t-St.)**
MS**	111/112 (99.1)		25.1	118/121 (97.5)			25.6	n.s.		2.8 (0.3-71.4)		n.s.
**HC**	107/121 (88.4)		24.3	69/73 (94.5)			23.1	n.s.		2.3 (0.7-8.5)		n.s.
p***	0.0009		0.00005	n.s.			<0.00001					
**OR (95% CI)**	14.5 (2.0-301.1)			2.3 (0.4-13.3)								
**B. VCA IgG**												
	HLA DRB1*15:01				NO HLA DRB1*15:01							
	**n/N (%)**	Titers*			**n/N (%)**	Titers*			**p (Chi)**	**OR (95% CI)**	**p (t-St.)**	
MS**	120/120 (100)	60.8			194/195 (99.5)	59.0			n.s.	–	n.s.	
**HC**	42/44 (95.5)	60.8			215/226 (95.1)	54.7			n.s.	1.1 (0.2-7.3)	n.s.	
p***	0.018	0.02			0.007	0.00008						
**OR (95% CI)**	–				9.9 (1.3-207.6)							
	**CURRENT SMOKER**				**NO SMOKER**							
	**n/N (%)**	Titers*			**n/N (%)**	Titers*			**p (Chi)**	**OR (95% CI)**	**p (t-St.)**	
MS**	112/112 (100)	61.5			134/134 (100)	58.6			n.s.	–	n.s.	
**HC**	72/73 (98.6)	58.5			202/216 (93.5)	54.7			n.s.	5.0 (0.7-103.5)	n.s.	
p***	n.s.	n.s.			0.003	0.0007						
**OR (95% CI)**	–				–							
	25(OH)D< MVS****				25(OH)D> MVS****							
	**n/N (%)**	Titers*			**n/N (%)**	Titers*			**p (Chi)**	**OR (95% CI)**	**p (t-St.)**	
MS**	112/112 (100)	61.0			121/121 (100)	60.0			n.s.	–	n.s.	
**HC**	115/121 (95.0)	56.2			71/73 (97.3)	56.3			n.s.	1.9 (0.3-13.7)	n.s.	
p***	0.017	0.005			n.s.	0.0003						
**OR (95% CI)**	–				–							

*Median values of the arbitrary units (AU). **Serologies performed in samples collected before DMT onset. ***Student t test (for titers) and Chi-square test (for categorical variables). **** MVS, Median value of each semester: 16.9 ng/ml for the first semester of the year and 24.0 ng/ml for the second semester of the year, in MS patients; 19.8 ng/ml and 24.7 ng/ml, respectively, in healthy controls. n.s., not significant.

### EBNA-1 and VCA IgG antibodies in MS patients prior DMT onset and healthy controls: correlation with clinical and demographical variables

We did not find any statistical difference in relation to the gender, nor in the untreated MS samples nor in the healthy control group (**Supplementary Table 2**). Regarding the age, we found a correlation with the VCA IgG titers in the untreated MS samples (r=0.180; p=0.001) and in the control group (r=0.116; p=0.047; not significant after Bonferroni correction for multiple comparisons), but any correlation was not found with EBNA-1 (p=0.647 and p=0.393 for MS patients and controls, respectively).

We also analyzed possible correlations between EBNA-1 and VCA IgG titers with different clinical variables in the samples collected before DMT in the MS group (**Table 3**): we only found a negative correlation between EBNA-1 IgG titers and the number of relapses two-years before starting DMTs (r= –0.153; p=0.006). Lastly, we stratified these variables according to smoking habit, vitamin D levels and HLA DRB1*15:01: VCA IgG titers were increased in smoker female MS patients in comparison with non-smoker female MS patients (p=0.001); VCA IgG titers correlated with ARR in non-smoker MS patients (r=–0.371; p=0.002); finally, VCA IgG titers correlated with the age in non-smoker MS patients (r=0.364; p=0.003) and in non-carriers of HLA-DRB1*15:01 MS patients (r=0.220; p=0.002).

**Table 3:** Correlations between EBNA-1 and VCA IgG titers with different clinical variables in the samples collected before DMT onset in the MS group.

		Starting Age	Disease duration	EDSS	Relapses 2-years before DMT onset	ARR
**EBNA-1 IgG titers**	**r**	0.131	–0.116	–0.107	–0.153	–0.073
	**p**	0.018	0.036	n.s.	**0.006**	n.s.
**VCA IgG titers**	**r**	0.113	0.067	0.075	–0.106	–0.102
	**p**	0.041	n.s.	n.s.	n.s.	n.s.

Correlations were assessed by using the Spearman’s rank correlation coefficient (r). Bold values indicates the statistically significant values after Bonferroni correction (p<0.007); significant p values prior Bonferroni correction are also shown. ARR, annualized relapse rate (number of relapses since the beginning of the disease/duration of the disease [years]). n.s., not significant.

### EBNA-1 and VCA IgG titers before DMT onset was not associated with the clinical response after two-years of follow-up

As we can see in **Table 4**, we did not find any statistical significant association between EBNA-1 and/or VCA IgG levels at the baseline visit (before DMT onset) and the progression of the disease, the number of relapses, the clinical response or the therapeutic failure, after two years of follow-up with the different DMTs. Stratification according to smoking habit, vitamin D levels and HLA DRB1*15:01 did not yield any statistical significant difference for any clinical variable analyzed (data not shown).

**Table 4:** EBNA-1 and VCA IgG titers at baseline visit (prior DMT onset) and progression, number of relapses, clinical response and therapeutic failure after two years of follow-up with DMT.

	EBNA-1 IgG		VCA IgG	
	**PROGRESSION**		**PROGRESSION**	
	**pos./N (%)**	Titers*	**pos./N (%)**	Titers*
**YES**	51/53 (96.2)	25.7	53/53 (100)	59.1
**NO**	265/270 (98.1)	25.3	269/270 (99.6)	59.9
p**	n.s.	n.s.	n.s.	n.s.
**OR (95% CI)**				
	**RELAPSES**		**RELAPSES**	
	**pos./N (%)**	Titers*	**pos./N (%)**	Titers*
**YES**	128/131 (97.7)	25.5	130/131 (99.2)	60.1
**NO**	190/194 (97.9)	25.3	194/194(100)	59.4
p**	n.s.	n.s.	n.s.	n.s.
**OR (95% CI)**				
	**CLINICAL RESPONSE**		**CLINICAL RESPONSE**	
	**pos./N (%)**	Titers*	**pos./N (%)**	Titers*
**YES**	165/168 (98.2)	25.3	168/168 (100)	59.7
**NO**	153/157 (97.5)	25.5	156/157 (99.4)	59.9
p**	n.s.	n.s.	n.s.	n.s.
**OR (95% CI)**				
	**THERAPEUTIC FAILURE**		**THERAPEUTIC FAILURE**	
	**pos./N (%)**	Titers*	**pos./N (%)**	Titers*
**YES**	92/94 (97.9)	25.6	94/94 (100)	59.0
**NO**	226/231 (97.8)	25.2	230/231 (99.6)	60.1
p**	n.s.	n.s.	n.s.	n.s.
**OR (95% CI)**				

*Median values of the arbitrary units (AU). **Student t test (for titers) and Chi-square test (for categorical variables). n.s., not significant.

### EBNA-1 and VCA IgG prevalence and titers did not change significantly after two years follow-up with DMTs

As we can see in **Table 5**, neither the prevalence nor the titers of IgG antibodies against EBNA-1 and VCA changed significantly after two years of treatment. We did not find differences either after stratification according to smoking habit, vitamin D levels and HLA DRB1*15:01 (data not shown).

**Table 5:** Prevalences and median titers of the anti-EBNA-1 and VCA IgG antibodies before and after 2-years of treatment with the different DMTs.

	Anti-EBNA-1 IgG					
**Prevalences**			Median Titers (AU)*		
**DMTs**	**Before**	**2-years**	**p**	**Before**	**2-years**	**p**
TOTAL	97.8% (318/325)	98.5% (320/325)	n.s.	25.4	25.7	n.s.
IFN-beta	96.9% (127/131)	97.7% (128/131)	n.s.	25.9	26.2	n.s.
GA	98.8% (82/83)	98.8% (82/83)	n.s.	25.4	26.0	n.s.
NTZ	98.2% (109/111)	99.1% (110/111)	n.s.	24.9	24.9	n.s.
**Anti-VCA IgG**						
	**Prevalences**			Median Titers (AU)*		
**DMTs**	**Before**	**2-years**	p**	**Before**	**2-years**	p**
TOTAL	99.7% (324/325)	99.4% (323/325)	n.s.	59.9	60.7	n.s.
IFN-beta	99.2% (130/131)	99.2% (130/131)	n.s.	58.6	59.8	n.s.
GA	100% (83/83)	100% (83/83)	n.s.	59.0	61.5	n.s.
NTZ	100% (111/111)	99.1% (110/111)	n.s.	61.1	61.6	n.s.

TOTAL: prevalence and median titers of all the different disease modifying therapies (DMTs); IFN-beta, interferon beta; GA, glatiramer acetate; NTZ, natalizumab. *Median values of the arbitrary units (AU). **Student t test (for titers) and Chi-square test (for categorical variables). n.s., not significant.

### EBNA-1 and VCA IgG titers variation after two years follow-up with DMTs did not correlate with the clinical response

We did not find any statistical correlation in the variation of the EBNA-1 and VCA IgG titers between the basal and the 24 month samples and the number of relapses, the progression of the disease, the clinical response, the NEDA-3 condition or the therapeutic failure to any of the DMTs included in the study. The stratification according to smoking habit, vitamin D levels and HLA DRB1*15:01 did not yield any significant correlation (data not shown).

### EBNA-1 and VCA IgG titers before DMT onset were associated with different HLA alleles

**Figure 1:**
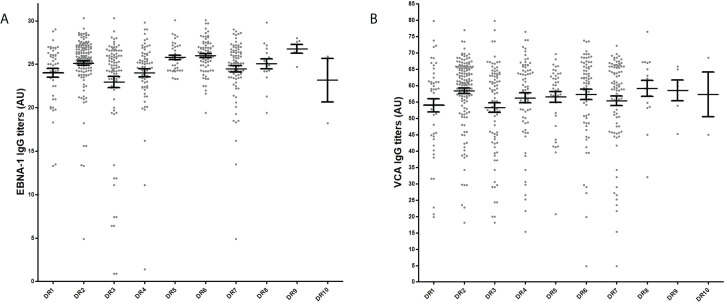
EBNA-1 **(A)** and VCA **(B)** IgG titres in serum samples of MS patients prior DMT treatment according to HLA-DR. Grey points correspond to EBNA-1 and VCA IgG values in AU of each sample; in black, mean plus standard error of the mean for each HLA-DR.

In **Figure 1** we can see the distribution of EBNA-1 and VCA IgG titers according to the HLA-DR of the MS patients included in the study. After Bonferroni corrections for multiple comparisons, we found the following statistical associations: 1) HLA-DR3+ MS patients and lower EBNA-1 IgG titers: 14/143 (9.8%) MS patients with the highest titers were HLA-DR3+ vs. 30/143 (21.0%) among those with the lowest titers (p=0.009; O.R. = 2.5). 2) HLA-DR6+ MS patients and higher EBNA-1 IgG titers: 29/143 (20.3%) MS patients with the highest titers belonged were HLA-DR6+ vs. 11/143 (7.7%) among those with the lowest titers (p=0.002; O.R. = 3.1). We did not find any statistical association between HLA-DR and VCA IgG titers. Statistical associations for HLA-DQA and -DQB are shown in **Supplementary Tables 3, 4**.

### EBNA-1 and VCA IgG titers before DMT onset and SNPs from GWAS

After Bonferroni correction for multiple comparisons, we found a statistical association between rs11129295 (located on chromosome 3 - EOMES gene) and EBNA-1 IgG titers: 25.7 AU, 25.3 AU and 24.2 AU were the median values of the EBNA-1 IgG titers for TT, CT and CC genotypes (p=0.0008. Kruskal Wallis test) (p values for all the genotype and allele comparisons are in **Supplementary Table 5, 6**).

### Epidemiological and clinical data of MS patients according to the genetic background associated with EBNA-1 IgG titers

According to the genetic associations found, we compared different epidemiological and clinical data between DR3+/DR6-/rs11129295TT- MS patients and the rest of the patients. As we can see in **Table 6**, we found a statistical significant difference for the starting age of the disease: MS started 3.5 years later among those MS patients with genetic factors associated with lower EBNA-1 IgG titers.

**Table 6:** Epidemiological, clinical and serological data of MS patients before DMT onset according to the genetic background associated with EBNA-1 IgG titers.

EPIDEMIOLOGICAL AND CLINICAL DATA						
	n	Gender (F/M)	Age	Starting Age	MSSS	ARR
**DR3+/DR6-/rs11129295TT-**	40	26/14	36	31.5	2.98	1.1
**Rest of MS patients**	247	168/79	36	28.0	3.34	1.0
p value*		n.s.	n.s.	**0.006**	n.s.	n.s.
**SEROLOGICAL DATA**						
	**n**	**EBNA-1 IgG titers (AU)**	**EBNA-1 IgG % pos. (n/N)**	**VCA IgG titers (AU)**	**VCA IgG % pos. (n/N)**	
**DR3+/DR6-/rs11129295TT-**	40	24.7	95.0% (38/40)	56.5	100% (40/40)	
**Rest of MS patients**	247	25.4	98.8% (244/247)	60.2	99.6% (246/247)	
p value*		**0.009**	n.s.	n.s.	n.s.	

*Student t test (for continuous variables) and Chi-square test (for categorical variables). Median values for continuous variables. n.s., not significant; MSSS, Multiple sclerosis severity score (before DMT onset). ARR, annualized relapse rate (since the beginning of the disease until DMT onset); AU, arbitrary units; % pos., percentage of positive samples. Bold values indicates the statistically significant values.

## Discussion

We found statistical significant differences in EBNA-1 and VCA IgG prevalences and titers between MS patients and controls. Furthermore, all MS patients were positive for EBV antibodies (EBNA-1 and/or VCA) but not the controls (94.9%, p=0.00004). Thus, these results confirm that MS occurs rarely in absence of EBV as it has been previously suggested (6). After stratification by other predisposing factors for MS like HLA DRB1*15:01, vitamin D deficiency and smoking habit, EBNA-1 and VCA IgG prevalences and titers remained significantly higher in MS patients than in controls. A recent study with the Swedish cohort showed that smokers had higher EBNA-1 antibody levels than never smokers; authors suggested that smoking habit and EBNA-1 antibody levels could act synergistically to increase MS risk (16). Interestingly, we did not find a significant difference in EBNA-1 and VCA IgG prevalences between current smokers (MS patients and controls); a higher prevalence of both antibodies was found in the control group of current smokers than in the control group of no smokers, although this increase was not higher enough to be significantly different between both groups. Regarding vitamin D, MS and EBV, it has been published that high-dose vitamin D supplementation reduces anti-EBNA-1 antibody levels in MS patients (17); also, EBV viral load measured by quantitative PCR was significantly higher when vitamin D levels were low, demonstrating an inverse correlation between vitamin D and EBV viral load (18). Furthermore, a previous study showed monthly differences in EBNA-1 IgG levels and an association between EBNA-1 IgG, vitamin D levels and HLA-DRB1*15 indicating that EBNA-1 IgG serum levels could be affected by genetic and environmental factors (19). In our study, we did not find significant differences in EBNA-1 or VCA IgG prevalences or titers in relation to HLA-DRB1*15:01 or the vitamin D levels nor in MS group nor in control group.

When we analyzed possible correlations between EBV IgG antibodies and clinical and demographic variables prior DMT onset, we found a positive significant correlation between VCA IgG titers and the age of MS patients (but with a very low correlation coefficient). A previous study with Lebanese MS patients also found that older age was associated with a higher anti-VCA titer (20). Similarly, we found an inverse low correlation (although significant) between EBNA-1 IgG titers and the annualized relapse rate two years prior recruitment. Prior results also suggested that anti-EBNA-1 IgG levels in MS could not be a reliable marker of MS clinical disease activity (21, 22). However, both data should be analyzed in further studies with wider cohorts to better understand their possible clinical relevance.

In this study we also analyzed the possible association between the EBNA-1 and VCA IgG variation after two years of follow-up with the clinical response to different DMTs in the same period. However, we did not find any relation between EBV IgG antibodies variation and the clinical parameters analyzed between the basal visit (without treatment) and the 24 month visit (after two years with different DMT). Previous articles found similar results. Raffel et al. (23) investigated the effect of interferon-beta and natalizumab therapy on prospective sera anti-EBNA-1 IgG titres over 12 months of treatment; for both, there was no significant difference between pre-therapy and post-therapy anti-EBNA-1 IgG titres. In a study of Comabella et al. (24), they evaluated cellular and humoral immune responses to EBV encoded antigens in patients with MS before and 1 year after interferon-beta treatment by ELISA and flow cytometry; although clinically effective interferon-beta therapy was associated with a downregulation of proliferative T cell responses to the latent EBNA-1, EBNA1-specific IgG responses as well as cellular and humoral immune responses to MHC class I restricted EBV antigens expressed during lytic replication and viral B cell transformation were similar before and after interferon-beta therapy. Therefore, it seems that these treatments would not have an effect on EBV antibody titers. This is probably related to their mechanisms of action and with the cell populations that could be modified by them. But, what about those new MS therapies that have B-cells as primary target? The emerging B-cell depleting therapies, particularly anti-CD20 monoclonal antibodies, would deplete the primary site of EBV latent infection. Thus, it has been suggested that the high efficacy described for these treatments (25, 26) could be associated, at least in part, with their effects on EBV. There are not previous studies with these therapies on EBV viral load or antibody titers in MS patients. However, a study with a small cohort of MS patients that initiated ocrelizumab showed that this treatment decreases the cellular immune response to EBV as measured by *in vitro* proliferation and IFN-gamma secretion (27). In another publication, a 56-year-old woman with EBV-associated posttransplant lymphoproliferative disorder following allogeneic hematopoietic stem cell transplantation was treated with ofatumumab, with a significant decrease in EBV viral load (28). Therefore, it would be of great interest to perform longitudinal studies with these therapies to analyze the possible correlation between the clinical response and the variation in the EBV viral loads or antibody titers.

Finally, we also analyzed the possible association between the EBNA-1 and VCA IgG titers with the genetic background of the MS patients in serum samples collected when they were untreated. As we can see in the results section, we found several significant associations: HLA-DR3+ with lower EBNA-1 IgG titers, HLA-DR6+ with higher EBNA-1 IgG titers and rs11129295TT carriers with higher EBNA-1 IgG titers. Prior studies have found different significant correlations between HLA alleles and EBV viral load and antibody titers in different MS cohorts; thus, it has been suggested that the mechanism through which HLA genes would influence the risk of MS may, at least in part, involve the immune control of EBV infection (29, 30). This is the first study performed in Spanish MS patients that evaluate the possible interaction between different genetic factors and the levels of EBNA-1 and VCA IgG titers. Beside the HLA alleles cited above, we also found a statistical significant association for the rs11129295TT genotype and rs11129295T allele. This SNP is located on chromosome 3 and is associated with the expression of the transcription factor EOMES which is induced in effector CD8+ T cells *in vitro* and *in vivo* (31). Furthermore, CD8+ T cells deficient in this transcription factor fail to differentiate into functional killers (32); also, mature NK cells from which EOMES was deleted reverted to phenotypic immaturity (33). In MS patients it has been described that EOMES expression is significantly lower than in healthy controls (34); that could mean a worse control of infections, like EBV, in MS. An analysis of twins from the Brisbane Systems Genetics Study determined that the heritability of EOMES was 0.48, although it could be underestimate; besides, EOMES expression was normalized to healthy control levels with MS therapies like natalizumab (35). In our study, when we compared different epidemiological and clinical variables between those with genetic factors associated with lower EBNA-1 IgG titers and all other MS patients, we found MS started 3.5 years later among the first. Previously, the HLA burden, especially the DRB1*1501 allele, had been associated on average to a younger age at onset (36, 37). In our Spanish cohort of MS patients, we found that the combination of different HLA alleles and a no-HLA SNP could be associated with a different MS phenotype that should be further studied.

Among the limitations of the study, it has to be considered the absence of HLA class I data; given the result on EOMES, a transcription factor that may affect the CD8 response, genotyping for HLA class I would have been of interest. In addition, it would be very interesting to study the expression levels of EOMES and try to correlate them with the EBNA-1 titers in a longitudinal study like this and also to analyze the expression levels of this transcription factor according to EOMES genotyping.

In conclusion, these results confirm that MS occurs rarely in absence of EBV. Although other predisposing factors could be involved in MS pathology, prevalences and titers remained significantly higher in MS patients than in controls after stratification by them. Besides, an intriguing association between genetic burden and lower EBNA-1 IgG titers was associated with an earlier age of disease onset. Finally, the DMTs included in this study did not change significantly the titers of the anti-EBV antibodies analyzed; similar studies with B-cell–targeted therapies are needed.

## Data availability statement

The datasets presented in this study can be found in online repositories. The names of the repository/repositories and accession number(s) can be found in the article/**Supplementary Material**.

## Ethics statement

The studies involving human participants were reviewed and approved by Comité Ético de Investigación Clínica del Hospital Clínico San Carlos and Comité de Ética de la Investigación del Hospital Fundación Jiménez Díaz. The patients/participants provided their written informed consent to participate in this study.

## Author contributions

MD-M, LL-L and RA-L made substantial contributions to the conception or design of the work, the acquisition, analysis, and interpretation of data for the work, and drafting the work or revising it critically for important intellectual content. SP-P performed analysis, and interpretation of data for the work, and drafting the work or revising it critically for important intellectual content. AG-M took part in the acquisition of the data. MT took part in the acquisition of the data and drafting the work or revising it critically for important intellectual content. RA contributed drafting the work or revising it critically for important intellectual content. All authors contributed to the article and approved the submitted version.

## Funding

This work was financially supported by Instituto de Salud Carlos III (ISCIII)-Fondo Europeo de Desarrollo Regional (Feder) (PI18/00204), “REEM: Red Española de Esclerosis Múltiple” (RD16/0015/0013) and “Fundación LAIR”.

## Conflict of interest

RA has been a speaker or has participated in the advisory board of Novartis, Teva, Roche, Bristol, Janssem, Biogen, Merck and Sanofi-Genzyme. RA-L has received support for attending meetings from Biogen, Novartis and Sanofi-Genzyme.

The remaining authors declare that the research was conducted in the absence of any commercial or financial relationships that could be construed as a potential conflict of interest.

## Publisher’s note

All claims expressed in this article are solely those of the authors and do not necessarily represent those of their affiliated organizations, or those of the publisher, the editors and the reviewers. Any product that may be evaluated in this article, or claim that may be made by its manufacturer, is not guaranteed or endorsed by the publisher.
