# Supplementary material for: Epstein-Barr Virus and multiple sclerosis in a Spanish cohort: A two-years longitudinal study
Source: Front Immunol. 2022 Sep 14;13:991662. doi: 10.3389/fimmu.2022.991662 (PMC9515943; doi:10.3389/fimmu.2022.991662)
Supplement: Supplementary file 1 [file Table_1.docx]

Supplementary Material

**Supplementary Table 1.** SNPs selected from GWAS studies.

| **SNP** | **GENE** | **MAF*** |
| --- | --- | --- |
| rs10201872 (C/T) | SP140 | T=0.100/217 |
| rs10466829 (A/G) | CLECL1 | A=0.498/1084 |
| rs11129295 (C/T) | EOMES | C= 0.494/1076 |
| rs11154801 (A/C) | MYB | A=0.287/626 |
| rs11810217 (C/T) | EVI5 | T=0.153/334 |
| rs12212193 (A/G) | BACH2 | G=0.300/653 |
| rs12368653 (A/G) | CYP27B1 | A=0.317/690 |
| rs12466022 (A/C) | Unknown | A=0.304/663 |
| rs1250550 (G/T) | ZMIZ1 | T=0.295/643 |
| rs13192841 (A/G) | Unknown | A=0.189/411 |
| rs13333054 (C/T) | IRF8 | T= 0.302/657 |
| rs1335532 (C/T) | CD58 / LFA-3 | C=0.382/832 |
| rs140522 (A/G) | SCO2 | A=0.376/819 |
| rs1520333 (C/T) | IL7 | C=0.415/904 |
| rs17066096 (A/G) | IL22RA2 | G=0.172/375 |
| rs17174870 (C/T) | MERTK | T=0.218/475 |
| rs1738074 (A/G) | TAGAP | A=0.441/961 |
| rs2019960 (C/T) | PVT1 | C=0.243/530 |
| rs2119704 (A/C) | GALC | A=0.129/280 |
| rs2243123 (C/T) | IL12A | C=0.198/431 |
| rs2248359 (C/T) | CYP24A1 | T=0.439/956 |
| rs2283792 (G/T) | MAPK1 | T=0.459/1000 |
| rs2293370 (C/T) | CD80 | T=0.210/458 |
| rs2300603 (C/T) | BATF | C=0.221/481 |
| rs22303759 (G/T) | CD37 | G=0.270/588 |
| rs2546890 (A/G) | IL12B | A=0.433/944 |
| rs3118470 (C/T) | IL2RA | C=0.319/694 |
| rs4410871 (C/T) | MYC | T=0.298/650 |
| rs4613763 (C/T) | PTGER4 | C=0.110/239 |
| rs4648356 (A/C) | MMEL1 | A=0.420/914 |
| rs4902647 (C/T) | ZFP36L1 | C=0.497/1083 |
| rs669607 (G/T) | Unknown | T=0.389/847 |
| rs7200786 (A/G) | CLEC16A | A=0.492/1072 |
| rs7238078 (G/T) | MALT1 | G=0.226/492 |
| rs7522462 (A/G) | KIF21B | A=0.150/326 |
| rs7595037 (C/T) | PLEK | C=0.352/767 |
| rs7923837 (A/G) | HHEX | A=0.427/930 |
| rs802734 (C/T) | THEMIS | C=0.198/432 |
| rs8112449 (A/G) | TYK2 | A=0.351/765 |
| rs874628 (C/T) | MPV17L2 | C=0.192/418 |
| rs9282641 (A/G) | CD86 | A=0.050/109 |
| rs949143 (A/G) | ARL6IP4 | G=0.462/1007 |
| * Minor allele frequency | | |

**Supplementary Table 2.** EBNA-1 and VCA IgG prevalence and titers in relation to the gender in MS patients and healthy controls.

| **GENDER** | **MS PATIENTS BEFORE DMT ONSET*** | | | | **HEALTHY CONTROLS** | | | |
| --- | --- | --- | --- | --- | --- | --- | --- | --- |
|  | **EBNA-1** | | **VCA** | | **EBNA-1** | | **VCA** | |
|  | **Prevalence** | **Titers**** | **Prevalence** | **Titers** | **Prevalence** | **Titers** | **Prevalence** | **Titers** |
| **Female** | 214/217 (98.6%) | 25.3 AU | 216/217  (99.5%) | 60.2 AU | 103/115  (89.6%) | 22.7 AU | 109/115  (94.8%) | 55.1 AU |
| **Male** | 104/108  (96.3%) | 25.4 AU | 108/108  (100%) | 59.0 AU | 154/180  (85.6%) | 24.6 AU | 170/180  (94.4%) | 55.8 AU |
| **p value***** | 0.175 | 0.625 | 0.480 | 0.431 | 0.316 | 0.866 | 0.900 | 0.675 |
| **O.R.****** | 2.7  (0.5-15.8) | - | - | - | 1.5  (0.7-3.2) |  | 1.1  (0.4-3.4) |  |

* Only samples collected in MS patients before starting DMTs. ** Median value. *** p-values were calculated from Chi-square test/Fisher’s exact test (for prevalence comparison) and Student’s t test (for the comparison of the IgG titers). **** Odds Ratios (OR) with the 95% Confidence Intervals (CI).

**Supplementary Table 3.** HLA-DQA frequency according to EBNA-1 and VCA IgG titers in serum samples of MS patients prior DMT treatment.

A.

| **DQA** | **EBNA-1 IgG titers** | | | | **% Q1** | **%**  **Q2** | **% >med.** | | **% Q3** | | **% Q4** | | **% <med.** | **p***  **Q1vsQ4** | **OR (95%CI) Q1vsQ4** | | **p***  **>Med<** | | **OR (95%CI) >Med<** |
| --- | --- | --- | --- | --- | --- | --- | --- | --- | --- | --- | --- | --- | --- | --- | --- | --- | --- | --- | --- |
|  | **Q1** | **Q2** | **Q3** | **Q4** |  |  |  |  |  |  |  |  |  |  |  |  |  |  |  |
| 0101 | 16 | 15 | 15 | 19 | 24.6% | 23.1% | 47.7% | | 23.1% | | 29.2% | | 52.3% | 0.603 | 0.8 (0.4-1.8) | | 0.715 | | 0.9 (0.5-1.6) |
| 0102 | 46 | 46 | 34 | 31 | 29.3% | 29.3% | 58.6% | | 21.7% | | 19.7% | | 41.4% | 0.041 | 1.7 (1.0-3.1) | | 0.010 | | 1.6 (1.1-2.4) |
| 0103 | 5 | 5 | 5 | 3 | 27.8% | 27.8% | 55.6% | | 27.8% | | 16.7% | | 44.4% | 0.467 | 1.7 (0.4-9.2) | | 0.621 | | 1.3 (0.5-3.6) |
| 0201 | 18 | 19 | 22 | 24 | 21.7% | 22.9% | 44.6% | | 26.5% | | 28.9% | | 55.4% | 0.327 | 0.7 (0.4-1.5) | | 0.301 | | 0.8 (0.5-1.3) |
| 0301 | 15 | 15 | 20 | 24 | 20.3% | 20.3% | 40.5% | | 27.0% | | 32.4% | | 59.5% | 0.126 | 0.6 (0.3-1.2) | | 0.086 | | 0.7 (0.4-1.1) |
| 0302 | 0 | 0 | 0 | 1 | 0.0% | 0.0% | 0.0% | | 0.0% | | 100.0% | | 100.0% | 0.318 | - | | 0.319 | | - |
| 0401 | 4 | 3 | 7 | 3 | 23.5% | 17.6% | 41.2% | | 41.2% | | 17.6% | | 58.8% | 0.695 | 1.4 (0.3-7.8) | | 0.469 | | 0.7 (0.2-2.0) |
| 0501 | 35 | 36 | 36 | 35 | 24.6% | 25.4% | 50.0% | | 25.4% | | 24.6% | | 50.0% | 0.972 | 1.0 (0.6-1.8) | | 0.961 | | 1.0 (0.7-1.5) |
| **DQA** | **VCA IgG titers** | | | | **% Q1** | **%**  **Q2** | | **% >med.** | | **% Q3** | | **% Q4** | **% <med.** | **p***  **Q1vsQ4** | **OR (95%CI) Q1vsQ4** | **p***  **>Med<** | | **OR (95%CI) >Med<** | |
|  | **Q1** | **Q2** | **Q3** | **Q4** |  |  |  |  |  |  |  |  |  |  |  |  |  |  |  |
| 0101 | 15 | 11 | 15 | 24 | 23.1% | 16.9% | | 40.0% | | 23.1% | | 36.9% | 60.0% | 0.126 | 0.6 (0.3-1.2) | 0.092 | | 0.6 (0.4-1.1) | |
| 0102 | 47 | 48 | 37 | 25 | 29.9% | 30.6% | | 60.5% | | 23.6% | | 15.9% | 39.5% | **0.002** | **2.4 (1.3-4.3)** | **0.002** | | **1.8 (1.2-2.7)** | |
| 0103 | 7 | 2 | 4 | 5 | 38.9% | 11.1% | | 50.0% | | 22.2% | | 27.8% | 50.0% | 0.547 | 1.4 (0.4-5.4) | 0.988 | | 1.0 (0.4-2.8) | |
| 0201 | 21 | 16 | 21 | 25 | 25.3% | 19.3% | | 44.6% | | 25.3% | | 30.1% | 55.4% | 0.536 | 0.8 (0.4-1.6) | 0.301 | | 0.8 (0.5-1.3) | |
| 0301 | 18 | 18 | 19 | 19 | 24.3% | 24.3% | | 48.6% | | 25.7% | | 25.7% | 51.4% | 0.878 | 1.0 (0.5-2.0) | 0.829 | | 1.0 (0.6-1.6) | |
| 0302 | 0 | 1 | 0 | 0 | 0.0% | 100.0% | | 100.0% | | 0.0% | | 0.0% | 0.0% | - | - | 0.315 | | - | |
| 0401 | 5 | 3 | 7 | 2 | 29.4% | 17.6% | | 47.1% | | 41.2% | | 11.8% | 52.9% | 0.247 | 2.6 (0.4-19.5) | 0.819 | | 0.9 (0.3-2.6) | |
| 0501 | 26 | 40 | 36 | 40 | 18.3% | 28.2% | | 46.5% | | 25.4% | | 28.2% | 53.5% | 0.057 | 0.6 (0.3-1.1) | 0.356 | | 0.8 (0.6-1.3) | |

B.

| **DQA** | **Q1 Q1** | **%** | **Q4 Q4** | **%** | **p***  **Q1vsQ4** | **OR (CI95%) Q1vsQ4** |
| --- | --- | --- | --- | --- | --- | --- |
| 0101 | 5 | 9.6% | 9 | 20.5% | 0.134 | 0.4 (0.1-1.5) |
| 0102 | 21 | 40.4% | 6 | 13.6% | **0.004** | **4.3 (1.4-13.7)** |
| 0103 | 2 | 3.8% | 1 | 2.3% | 0.659 | 1.7 (0.1-49.8) |
| 0201 | 8 | 15.4% | 11 | 25.0% | 0.239 | 0.6 (0.2-1.7) |
| 0301 | 5 | 9.6% | 9 | 20.5% | 0.134 | 0.4 (0.1-1.5) |
| 0302 | 0 | 0.0% | 0 | 0.0% | - | - |
| 0401 | 2 | 3.8% | 0 | 0.0% | 0.189 | - |
| 0501 | 9 | 17.3% | 8 | 18.2% | 0.911 | 0.9 (0.3-3.0) |

| **DQA** | **>Med** | **%** | **<Med** | **%** | **p***  **>Med<** | **OR (95%CI) >Med<** |
| --- | --- | --- | --- | --- | --- | --- |
| 0101 | 13 | 8.8% | 22 | 14.8% | 0.110 | 0.6 (0.3-1.2) |
| 0102 | 55 | 37.2% | 28 | 18.8% | **0.0004** | **2.6 (1.5-4.5)** |
| 0103 | 8 | 5.4% | 7 | 4.7% | 0.781 | 1.2 (0.4-3.7) |
| 0201 | 19 | 12.8% | 29 | 19.5% | 0.121 | 0.6 (0.3-1.2) |
| 0301 | 15 | 10.1% | 25 | 16.8% | 0.094 | 0.6 (0.3-1.2) |
| 0302 | 0 | 0.0% | 0 | 0.0% | - | - |
| 0401 | 4 | 2.7% | 6 | 4.0% | 0.527 | 0.7 (0.2-2.7) |
| 0501 | 34 | 23.0% | 32 | 21.5% | 0.756 | 1.1 (0.6-2.0) |

* p-values were calculated from Chi-square test. OR (95%CI): Odds Ratios (OR) with the 95% Confidence Intervals. Q1: highest IgG titers. Q4: lowest IgG titers. Med.: median value. Bold values indicate the statistically significant values after Bonferroni correction (p<0.006); significant p values prior Bonferroni correction are also shown. Table 6B. shows HLA-DQA frequency in MS patients with higher EBNA-1 and VCA IgG titers (both in Q1 or both above median value) vs. lower titers (both in Q4 or both below median value).

**Supplementary Table 4.** HLA-DQB frequency according to EBNA-1 and VCA IgG titers in serum samples of MS patients prior DMT treatment.

A.

| **DQB** | **EBNA-1 IgG titers** | | | | **% Q1** | **%**  **Q2** | **% >med.** | **% Q3** | **% Q4** | **% <med.** | **p***  **Q1vsQ4** | **OR (95%CI) Q1vsQ4** | **p***  **>Med<** | **OR (95%CI) >Med<** |
| --- | --- | --- | --- | --- | --- | --- | --- | --- | --- | --- | --- | --- | --- | --- |
|  | **Q1** | **Q2** | **Q3** | **Q4** |  |  |  |  |  |  |  |  |  |  |
| 0201 | 14 | 23 | 26 | 32 | 14,7% | 24,2% | 38,9% | 27,4% | 33,7% | 61,1% | **0,004** | **0,4 (0,2-0,8)** | 0,018 | 0,6 (0,4-0,9) |
| 0202 | 14 | 16 | 18 | 18 | 21,2% | 24,2% | 45,5% | 27,3% | 27,3% | 54,5% | 0,452 | 0,8 (0,3-1,7) | 0,431 | 0,8 (0,5-1,4) |
| 0301 | 26 | 19 | 19 | 13 | 33,8% | 24,7% | 58,4% | 24,7% | 16,9% | 41,6% | 0,025 | 2,2 (1,0-4,8) | 0,110 | 1,5 (0,9-2,5) |
| 0302 | 10 | 7 | 16 | 21 | 18,5% | 13,0% | 31,5% | 29,6% | 38,9% | 68,5% | 0,036 | 0,4 (0,2-1,0) | **0,004** | **0,4 (0,2-0,8)** |
| 0303 | 5 | 5 | 2 | 1 | 38,5% | 38,5% | 76,9% | 15,4% | 7,7% | 23,1% | 0,099 | 5,2 (0,6-118,1) | 0,049 | 3,4 (0,9-15,8) |
| 0402 | 1 | 2 | 4 | 2 | 11,1% | 22,2% | 33,3% | 44,4% | 22,2% | 66,7% | 0,562 | 0,5 (0,0-7,1) | 0,313 | 0,5 (0,1-2,2) |
| 0501 | 10 | 13 | 9 | 16 | 20,8% | 27,1% | 47,9% | 18,8% | 33,3% | 52,1% | 0,216 | 0,6 (0,2-1,5) | 0,763 | 0,9 (0,5-1,7) |
| 0502 | 4 | 3 | 4 | 1 | 33,3% | 25,0% | 58,3% | 33,3% | 8,3% | 41,7% | 0,176 | 4,1 (0,4-97,3) | 0,559 | 1,4 (0,4-5,2) |
| 0503 | 6 | 3 | 4 | 1 | 42,9% | 21,4% | 64,3% | 28,6% | 7,1% | 35,7% | 0,056 | 6,2 (0,7-139,1) | 0,279 | 1,8 (0,6-6,4) |
| 0601 | 1 | 0 | 3 | 0 | 25,0% | 0,0% | 25,0% | 75,0% | 0,0% | 75,0% | 0,316 | - | 0,316 | 0,3 (0,0-3,6) |
| 0602 | 39 | 36 | 28 | 29 | 29,5% | 27,3% | 56,8% | 21,2% | 22,0% | 43,2% | 0,162 | 1,5 (0,8-2,7) | 0,072 | 1,4 (1,0-2,2) |
| 0603 | 4 | 5 | 2 | 3 | 28,6% | 35,7% | 64,3% | 14,3% | 21,4% | 35,7% | 0,702 | 1,3 (0,3-7,7) | 0,279 | 1,8 (0,6-6,4) |
| 0604 | 3 | 6 | 2 | 1 | 25,0% | 50,0% | 75,0% | 16,7% | 8,3% | 25,0% | 0,314 | 3,0 (0,3-76,9) | 0,080 | 3,1 (0,8-14,4) |
| 0609 | 1 | 0 | 1 | 0 | 50,0% | 0,0% | 50,0% | 50,0% | 0,0% | 50,0% | 0,316 | - | 1,000 | 1,0 (0,0-36,7) |

| **DQB** | **VCA IgG titers** | | | | **% Q1** | **%**  **Q2** | **% >med.** | **% Q3** | **% Q4** | **% <med.** | **p***  **Q1vsQ4** | **OR (95%CI) Q1vsQ4** | **p***  **>Med<** | **OR (95%CI) >Med<** |
| --- | --- | --- | --- | --- | --- | --- | --- | --- | --- | --- | --- | --- | --- | --- |
|  | **Q1** | **Q2** | **Q3** | **Q4** |  |  |  |  |  |  |  |  |  |  |
| 0201 | 15 | 26 | 24 | 30 | 15,8% | 27,4% | 43,2% | 25,3% | 31,6% | 56,8% | 0,015 | 0,4 (0,2-0,9) | 0,143 | 0,7 (0,5-1,2) |
| 0202 | 18 | 11 | 14 | 23 | 27,3% | 16,7% | 43,9% | 21,2% | 34,8% | 56,1% | 0,397 | 0,8 (0,4-1,5) | 0,294 | 0,8 (0,4-1,3) |
| 0301 | 17 | 22 | 22 | 16 | 22,1% | 28,6% | 50,6% | 28,6% | 20,8% | 49,4% | 0,853 | 1,1 (0,5-2,4) | 0,902 | 1,0 (0,6-1,7) |
| 0302 | 14 | 13 | 13 | 14 | 25,9% | 24,1% | 50,0% | 24,1% | 25,9% | 50,0% | 1,000 | 1,0 (0,4-2,3) | 1,000 | 1,0 (0,6-1,8) |
| 0303 | 4 | 5 | 3 | 1 | 30,8% | 38,5% | 69,2% | 23,1% | 7,7% | 30,8% | 0,176 | 4,1 (0,4-97,3) | 0,161 | 2,3 (0,6-9,0) |
| 0402 | 2 | 1 | 5 | 1 | 22,2% | 11,1% | 33,3% | 55,6% | 11,1% | 66,7% | 0,562 | 2,0 (0,1-56,8) | 0,313 | 0,5 (0,1-2,2) |
| 0501 | 11 | 7 | 14 | 16 | 22,9% | 14,6% | 37,5% | 29,2% | 33,3% | 62,5% | 0,311 | 0,7 (0,3-1,6) | 0,070 | 0,6 (0,3-1,1) |
| 0502 | 4 | 4 | 2 | 2 | 33,3% | 33,3% | 66,7% | 16,7% | 16,7% | 33,3% | 0,409 | 2,0 (0,3-16,2) | 0,238 | 2,1 (0,6-8,4) |
| 0503 | 2 | 2 | 2 | 8 | 14,3% | 14,3% | 28,6% | 14,3% | 57,1% | 71,4% | 0,053 | 0,2 (0,0-1,2) | 0,104 | 0,4 (0,1-1,4) |
| 0601 | 0 | 1 | 2 | 1 | 0,0% | 25,0% | 25,0% | 50,0% | 25,0% | 75,0% | 0,316 | - | 0,316 | 0,3 (0,0-3,6) |
| 0602 | 39 | 40 | 31 | 22 | 29,5% | 30,3% | 59,8% | 23,5% | 16,7% | 40,2% | 0,013 | 2,1 (1,1-3,9) | 0,009 | 1,7 (1,1-2,6) |
| 0603 | 7 | 1 | 3 | 3 | 50,0% | 7,1% | 57,1% | 21,4% | 21,4% | 42,9% | 0,198 | 2,4 (0,6-12,0) | 0,588 | 1,3 (0,4-4,4) |
| 0604 | 5 | 3 | 3 | 1 | 41,7% | 25,0% | 66,7% | 25,0% | 8,3% | 33,3% | 0,099 | 5,2 (0,6-118,1) | 0,243 | 2,0 (0,6-8,1) |
| 0609 | 0 | 2 | 0 | 0 | 0,0% | 100,0% | 100,0% | 0,0% | 0,0% | 0,0% | - | - | 0,157 | - |

B.

| **DQB** | **Q1 Q1** | **%** | **Q4 Q4** | **% 4** | **p***  **Q1vsQ4** | **OR (CI95%) Q1vsQ4** |
| --- | --- | --- | --- | --- | --- | --- |
| 0201 | 4 | 8,0% | 7 | 15,9% | 0,234 | 0,5 (0,1-2,0) |
| 0202 | 6 | 12,0% | 10 | 22,7% | 0,167 | 0,5 (0,1-1,6) |
| 0301 | 6 | 12,0% | 4 | 9,1% | 0,648 | 1,4 (0,3-6,3) |
| 0302 | 5 | 10,0% | 7 | 15,9% | 0,392 | 0,6 (0,2-2,3) |
| 0303 | 1 | 2,0% | 0 | 0,0% | 0,346 | - |
| 0402 | 0 | 0,0% | 0 | 0,0% | - | - |
| 0501 | 4 | 8,0% | 8 | 18,2% | 0,140 | 0,4 (0,1-1,6) |
| 0502 | 1 | 2,0% | 0 | 0,0% | 0,346 | - |
| 0503 | 1 | 2,0% | 1 | 2,3% | 0,927 | 0,9 (0,0-33,3) |
| 0601 | 0 | 0,0% | 0 | 0,0% | - | - |
| 0602 | 18 | 36,0% | 6 | 13,6% | 0,013 | 3,6 (1,2-11,5) |
| 0603 | 2 | 4,0% | 1 | 2,3% | 0,635 | 1,8 (0,1-51,9) |
| 0604 | 2 | 4,0% | 0 | 0,0% | 0,180 | - |
| 0609 | 0 | 0,0% | 0 | 0,0% | - | - |

| **DQB** | **>Med** | **%** | **<Med** | **%** | **p***  **>Med<** | **OR (95%CI) >Med<** |
| --- | --- | --- | --- | --- | --- | --- |
| 0201 | 15 | 10,2% | 32 | 21,8% | 0,007 | 0,4 (0,2-0,8) |
| 0202 | 13 | 8,8% | 20 | 13,6% | 0,196 | 0,6 (0,3-1,4) |
| 0301 | 24 | 16,3% | 17 | 11,6% | 0,239 | 1,5 (0,7-3,1) |
| 0302 | 9 | 6,1% | 19 | 12,9% | 0,047 | 0,4 (0,2-1,1) |
| 0303 | 8 | 5,4% | 2 | 1,4% | 0,054 | 4,2 (0,8-29,0) |
| 0402 | 1 | 0,7% | 4 | 2,7% | 0,176 | 0,2 (0,0-2,4) |
| 0501 | 10 | 6,8% | 17 | 11,6% | 0,157 | 0,6 (0,2-1,4) |
| 0502 | 6 | 4,1% | 3 | 2,0% | 0,310 | 2,0 (0,4-10,5) |
| 0503 | 3 | 2,0% | 4 | 2,7% | 0,702 | 0,7 (0,1-4,0) |
| 0601 | 1 | 0,7% | 3 | 2,0% | 0,314 | 0,3 (0,0-3,6) |
| 0602 | 44 | 29,9% | 22 | 15,0% | **0,002** | **2,4 (1,3-4,5)** |
| 0603 | 7 | 4,8% | 4 | 2,7% | 0,357 | 1,8 (0,5-7,4) |
| 0604 | 5 | 3,4% | 0 | 0,0% | 0,024 | - |
| 0609 | 1 | 0,7% | 0 | 0,0% | 0,316 | - |

* p-values were calculated from Chi-square test. OR (95%CI): Odds Ratios (OR) with the 95% Confidence Intervals. Q1: highest IgG titers. Q4: lowest IgG titers. Med.: median value. Bold values indicate the statistically significant values after Bonferroni correction (p<0.004); significant p values prior Bonferroni correction are also shown. Table 6B. shows HLA-DQB frequency in MS patients with higher EBNA-1 and VCA IgG titers (both in Q1 or both above median value) vs. lower titers (both in Q4 or both below median value).

**Supplementary Table 5.** EBNA-1 IgG titers association to genotypes and alleles from SNPs analysed.

| **SNP** | **GENE** | **MAF*** | **p value** EBNA-1 IgG titers / Host genotypes** | **p value*** EBNA-1 IgG titers / Host alleles** |
| --- | --- | --- | --- | --- |
| rs10201872 (C/T) | SP140 | T=0.100/217 | 0,1376 | 0,099 |
| rs10466829 (A/G) | CLECL1 | A=0.498/1084 | 0,4918 | 0,808 |
| rs11129295 (C/T) | EOMES | T= 0.494/1076 | **0,0008** | 0,007 |
| rs11154801 (A/C) | MYB | A=0.287/626 | 0,8503 | 0,776 |
| rs11810217 (C/T) | EVI5 | T=0.153/334 | 0,1880 | 0,013 |
| rs12212193 (A/G) | BACH2 | G=0.300/653 | 0,1938 | 0,574 |
| rs12368653 (A/G) | CYP27B1 | A=0.317/690 | 0,8176 | 0,336 |
| rs12466022 (A/C) | Unknown | A=0.304/663 | 0,9591 | 0,726 |
| rs1250550 (G/T) | ZMIZ1 | T=0.295/643 | 0,7869 | 0,156 |
| rs13192841 (A/G) | Unknown | A=0.189/411 | 0,9618 | 0,696 |
| rs13333054 (C/T) | IRF8 | T= 0.302/657 | 0,3059 | 0,202 |
| rs1335532 (C/T) | CD58 / LFA-3 | C=0.382/832 | 0,2714 | 0,889 |
| rs140522 (A/G) | SCO2 | A=0.376/819 | 0,4659 | 0,801 |
| rs1520333 (C/T) | IL7 | C=0.415/904 | 0,4799 | 0,700 |
| rs17066096 (A/G) | IL22RA2 | G=0.172/375 | 0,8398 | 0,857 |
| rs17174870 (C/T) | MERTK | T=0.218/475 | 0,4993 | 0,169 |
| rs1738074 (A/G) | TAGAP | A=0.441/961 | 0,5559 | 0,509 |
| rs2019960 (C/T) | PVT1 | C=0.243/530 | 0,5353 | 0,244 |
| rs2119704 (A/C) | GALC | A=0.129/280 | 0,1067 | 0,592 |
| rs2243123 (C/T) | IL12A | C=0.198/431 | 0,9485 | 0,879 |
| rs2248359 (C/T) | CYP24A1 | T=0.439/956 | 0,6224 | 0,949 |
| rs2283792 (G/T) | MAPK1 | T=0.459/1000 | 0,0118 | 0,099 |
| rs2293370 (C/T) | CD80 | T=0.210/458 | 0,8254 | 0,891 |
| rs2300603 (C/T) | BATF | C=0.221/481 | 0,5828 | 0,517 |
| rs22303759 (G/T) | CD37 | G=0.270/588 | 0,8936 | 0,903 |
| rs2546890 (A/G) | IL12B | A=0.433/944 | 0,2643 | 0,626 |
| rs3118470 (C/T) | IL2RA | C=0.319/694 | 0,5672 | 0,467 |
| rs4410871 (C/T) | MYC | T=0.298/650 | 0,9495 | 0,950 |
| rs4613763 (C/T) | PTGER4 | C=0.110/239 | 0,7957 | 0,868 |
| rs4648356 (A/C) | MMEL1 | A=0.420/914 | 0,7481 | 0,564 |
| rs4902647 (C/T) | ZFP36L1 | C=0.497/1083 | 0,5298 | 0,602 |
| rs669607 (G/T) | Unknown | T=0.389/847 | 0,5799 | 0,939 |
| rs7200786 (A/G) | CLEC16A | A=0.492/1072 | 0,4642 | 0,066 |
| rs7238078 (G/T) | MALT1 | G=0.226/492 | 0,7473 | 0,616 |
| rs7522462 (A/G) | KIF21B | A=0.150/326 | 0,2127 | 0,367 |
| rs7595037 (C/T) | PLEK | C=0.352/767 | 0,6402 | 0,129 |
| rs7923837 (A/G) | HHEX | A=0.427/930 | 0,1242 | 0,080 |
| rs802734 (C/T) | THEMIS | C=0.198/432 | 0,5067 | 0,735 |
| rs8112449 (A/G) | TYK2 | A=0.351/765 | 0,7609 | 0,955 |
| rs874628 (C/T) | MPV17L2 | C=0.192/418 | 0,3283 | 0,164 |
| rs9282641 (A/G) | CD86 | A=0.050/109 | 0,9527 | 0,398 |
| rs949143 (A/G) | ARL6IP4 | G=0.462/1007 | 0,6721 | 0,863 |

| * Minor allele frequency |
| --- |
| ** Kruskal Wallis test was performed to analyze EBNA-1 IgG titers between the three genotypes |
| *** Student's t-test was performed to analyze EBNA-1 IgG titers between both alleles of each SNP |
|  |
| In bold are those significant p values after Bonferroni correction for multiple comparisons (p<0,0012) |

**Supplementary Table 6.** VCA IgG titers association to genotypes and alleles from SNPs analysed.

| **SNP** | **GENE** | **MAF*** | **p value** VCA / Host genotypes** | **p value*** VCA / Host alleles** |
| --- | --- | --- | --- | --- |
| rs10201872 (C/T) | SP140 | T=0.100/217 | 0,014 | 0,651 |
| rs10466829 (A/G) | CLECL1 | A=0.498/1084 | 0,736 | 0,983 |
| rs11129295 (C/T) | EOMES | C= 0.494/1076 | 0,691 | 0,778 |
| rs11154801 (A/C) | MYB | A=0.287/626 | 0,255 | 0,381 |
| rs11810217 (C/T) | EVI5 | T=0.153/334 | 0,894 | 0,946 |
| rs12212193 (A/G) | BACH2 | G=0.300/653 | 0,674 | 0,156 |
| rs12368653 (A/G) | CYP27B1 | A=0.317/690 | 0,073 | 0,534 |
| rs12466022 (A/C) | Unknown | A=0.304/663 | 0,392 | 0,226 |
| rs1250550 (G/T) | ZMIZ1 | T=0.295/643 | 0,499 | 0,876 |
| rs13192841 (A/G) | Unknown | A=0.189/411 | 0,588 | 0,153 |
| rs13333054 (C/T) | IRF8 | T= 0.302/657 | 0,211 | 0,249 |
| rs1335532 (C/T) | CD58 / LFA-3 | C=0.382/832 | 0,587 | 0,688 |
| rs140522 (A/G) | SCO2 | A=0.376/819 | 0,343 | 0,165 |
| rs1520333 (C/T) | IL7 | C=0.415/904 | 0,362 | 0,449 |
| rs17066096 (A/G) | IL22RA2 | G=0.172/375 | 0,104 | 0,280 |
| rs17174870 (C/T) | MERTK | T=0.218/475 | 0,498 | 0,158 |
| rs1738074 (A/G) | TAGAP | A=0.441/961 | 0,229 | 0,257 |
| rs2019960 (C/T) | PVT1 | C=0.243/530 | 0,055 | 0,110 |
| rs2119704 (A/C) | GALC | A=0.129/280 | 0,814 | 0,528 |
| rs2243123 (C/T) | IL12A | C=0.198/431 | 0,303 | 0,197 |
| rs2248359 (C/T) | CYP24A1 | T=0.439/956 | 0,458 | 0,365 |
| rs2283792 (G/T) | MAPK1 | T=0.459/1000 | 0,828 | 0,846 |
| rs2293370 (C/T) | CD80 | T=0.210/458 | 0,310 | 0,937 |
| rs2300603 (C/T) | BATF | C=0.221/481 | 0,443 | 0,462 |
| rs22303759 (G/T) | CD37 | G=0.270/588 | 0,289 | 0,212 |
| rs2546890 (A/G) | IL12B | A=0.433/944 | 0,586 | 0,230 |
| rs3118470 (C/T) | IL2RA | C=0.319/694 | 0,375 | 0,511 |
| rs4410871 (C/T) | MYC | T=0.298/650 | 0,946 | 0,525 |
| rs4613763 (C/T) | PTGER4 | C=0.110/239 | 0,331 | 0,920 |
| rs4648356 (A/C) | MMEL1 | A=0.420/914 | 0,939 | 0,715 |
| rs4902647 (C/T) | ZFP36L1 | C=0.497/1083 | 0,618 | 0,289 |
| rs669607 (G/T) | Unknown | T=0.389/847 | 0,653 | 0,665 |
| rs7200786 (A/G) | CLEC16A | A=0.492/1072 | 0,264 | 0,974 |
| rs7238078 (G/T) | MALT1 | G=0.226/492 | 0,364 | 0,094 |
| rs7522462 (A/G) | KIF21B | A=0.150/326 | 0,822 | 0,905 |
| rs7595037 (C/T) | PLEK | C=0.352/767 | 0,610 | 0,600 |
| rs7923837 (A/G) | HHEX | A=0.427/930 | 0,896 | 0,657 |
| rs802734 (C/T) | THEMIS | C=0.198/432 | 0,963 | 0,865 |
| rs8112449 (A/G) | TYK2 | A=0.351/765 | 0,575 | 0,328 |
| rs874628 (C/T) | MPV17L2 | C=0.192/418 | 0,003 | 0,088 |
| rs9282641 (A/G) | CD86 | A=0.050/109 | 0,728 | 0,404 |
| rs949143 (A/G) | ARL6IP4 | G=0.462/1007 | 0,120 | 0,045 |

| * Minor allele frequency |
| --- |
| ** Kruskal Wallis test was performed to analyze EBNA-1 IgG titers between the three genotypes |
| *** Student's t-test was performed to analyze EBNA-1 IgG titers between both alleles of each SNP |
|  |
| In bold are those significant p values after Bonferroni correction for multiple comparisons (p<0,0012) |
